# Neurobiological Correlates in Internet Gaming Disorder: A Systematic Literature Review

**DOI:** 10.3389/fpsyt.2018.00166

**Published:** 2018-05-08

**Authors:** Daria J. Kuss, Halley M. Pontes, Mark D. Griffiths

**Affiliations:** International Gaming Research Unit, Psychology Department, Nottingham Trent University, Nottingham, United Kingdom

**Keywords:** Internet Gaming Disorder, IGD, fMRI, rsfMRI, VBM, PET, EEG, review

## Abstract

Internet Gaming Disorder (IGD) is a potential mental disorder currently included in the third section of the latest (fifth) edition of the Diagnostic and Statistical Manual for Mental Disorders (DSM-5) as a condition that requires additional research to be included in the main manual. Although research efforts in the area have increased, there is a continuing debate about the respective criteria to use as well as the status of the condition as mental health concern. Rather than using diagnostic criteria which are based on subjective symptom experience, the National Institute of Mental Health advocates the use of Research Domain Criteria (RDoC) which may support classifying mental disorders based on dimensions of observable behavior and neurobiological measures because mental disorders are viewed as biological disorders that involve brain circuits that implicate specific domains of cognition, emotion, and behavior. Consequently, IGD should be classified on its underlying neurobiology, as well as its subjective symptom experience. Therefore, the aim of this paper is to review the neurobiological correlates involved in IGD based on the current literature base. Altogether, 853 studies on the neurobiological correlates were identified on ProQuest (in the following scholarly databases: ProQuest Psychology Journals, PsycARTICLES, PsycINFO, Applied Social Sciences Index and Abstracts, and ERIC) and on MEDLINE, with the application of the exclusion criteria resulting in reviewing a total of 27 studies, using fMRI, rsfMRI, VBM, PET, and EEG methods. The results indicate there are significant neurobiological differences between healthy controls and individuals with IGD. The included studies suggest that compared to healthy controls, gaming addicts have poorer response-inhibition and emotion regulation, impaired prefrontal cortex (PFC) functioning and cognitive control, poorer working memory and decision-making capabilities, decreased visual and auditory functioning, and a deficiency in their neuronal reward system, similar to those found in individuals with substance-related addictions. This suggests both substance-related addictions and behavioral addictions share common predisposing factors and may be part of an addiction syndrome. Future research should focus on replicating the reported findings in different cultural contexts, in support of a neurobiological basis of classifying IGD and related disorders.

## Key concepts

***Functional Magnetic Resonance Imaging (fMRI)*** measures changes neuronal activity via levels of blood oxygen (BOLD) in the brain, as blood flow in “active” brain areas increases to transport more glucose, whilst transporting additional oxygenated hemoglobin molecules.

***Resting State Magnetic Resonance Imaging* (*rsfMRI*)** is a subtype of fMRI which measures blood oxygen levels (BOLD) to assess brain activity whilst the subject is in a resting state (i.e., not engaged in a specific activity). The aim is to investigate whether there are differences in brain function in individuals with particular conditions in comparison to healthy controls.

***Voxel-based morphometry (VBM)*** helps characterize subtle structural changes in the brain without the need of prior knowledge. This is especially important given videogame use can affect brain functioning in various ways that may result in changes at the behavioral and cognitive levels.

***Positron Emission Tomography (PET)*** measures metabolic activity in the brain by detecting gamma rays which are emitted through a tracer substance, which are then depicted through computer analysis.

Studies using ***Electroencephalography (EEG)*** are employed to detect neural activity from the underlying cortical areas (anterior, posterior, right, and left) in an individual's cerebral cortex using electrodes attached to the scalp. Using this technique, voltage fluctuations (i.e., current flow produced by excitation of neuronal synapses) are measured between pairs of electrodes.

## Introduction

*Internet Gaming Disorder* (IGD) is a potential mental disorder currently included in the third section of the latest (fifth) edition of the Diagnostic and Statistical Manual for Mental Disorders (DSM-5) as a condition that requires additional research to be included in the main manual (1). Although research efforts in the area have increased, there is a continuing debate about the respective criteria to use as well as the status of the condition as mental health concern [e.g., (2, 3)].

The controversies regarding the proposed classification of IGD in the DSM-5 concern the conceptual, theoretical, as well as methodological issues that have been raised by a number of scholars in the field. Firstly, it has been stated that the addiction framework is restricting because rather than being an addiction, problematic gaming may be the result of maladaptive coping and seeking to satisfy previously unmet needs (4). However, research (5) has also shown that dysfunctional coping and Internet addiction do not have to be mutually exclusive, but that the former predicts the latter, and may therefore suggest that gaming is a form of self-medication, and which is similar to other addictions (6). Secondly, it has been argued that if IGD results from other mental disorders it cannot be considered a bona fide addiction (7). However, from a clinical perspective, it is clear that comorbidity is the norm, not an exception, and this holds not just for Internet and gaming addiction (6, 8), but also for other psychopathology (9) including other addictions (6). Thirdly, previous research on IGD has been criticized for its methodological limitations, given that most research in the area has been conducted using non-clinical populations using psychometric (and therefore subjective) measures (10). However, there are increasing numbers of studies evaluating treatment-seeking clinical patients with IGD [e.g., (11–23)]. Furthermore, methodological limitations of research in the young field of IGD are limiting our understanding and generalization of findings, and therefore it is of utmost importance to continue researching the phenomenon both from a clinical perspective and using methods that can be considered more objective, such as assessing the neurobiological underpinnings of IGD.

Rather than assessing IGD subjectively by relying on diag-nostic criteria which are based on subjective symptom exp-erience, the National Institute of Mental Health (24) advocates the use of Research Domain Criteria (RDoC) which may support classifying mental disorders based on dimensions of observable behavior and neurobiological measures because mental disorders are viewed as biological disorders that involve brain circuits that implicate specific domains of cognition, emotion, and behavior. Consequently, IGD should be classified on its underlying neurobiology as well as its subjective symptom experience. Therefore, the aim of this paper is to review the neurobiological correlates in IGD based on the current literature base.

## Methods

Inclusion criteria used for the present review were: (i) assessing neurobiological mechanisms in IGD, (ii) empirical studies, (iii) using neuroimaging techniques, (iv) published in a peer-reviewed journal, (v) written in English, and (vi) published since 2012 as previous reviews have covered the timeframe before then (25). The database ProQuest was searched, including the following databases: Applied Social Sciences Index and Abstracts (ASSIA), ERIC, ProQuest Psychology Journals, PsycARTICLES, and PsycINFO, with another search performed on MEDLINE. The search included the most common types of neuroimaging techniques used in IGD research [i.e., electroencephalogram (EEG), positron emission tomography (PET), single-photon emission computed tomography (SPECT), functional magnetic resonance imaging (fMRI), structural magnetic resonance imaging (sMRI), diffusion-tensor imaging (DTI)] as reported in a previous systematic review [i.e., (25)], leading to the following search strategy: (patholog^*^ OR problem^*^ OR addict^*^ OR compulsive OR dependen^*^ OR disorder^*^) AND (video OR computer OR internet) gam^*^ AND (neuroimaging OR eeg OR pet OR spect or fmri OR smri OR dti). Each study's title and abstract were screened for eligibility. Full texts of all potentially relevant studies were then retrieved and further examined for eligibility.

## Results

**Figure 1:**
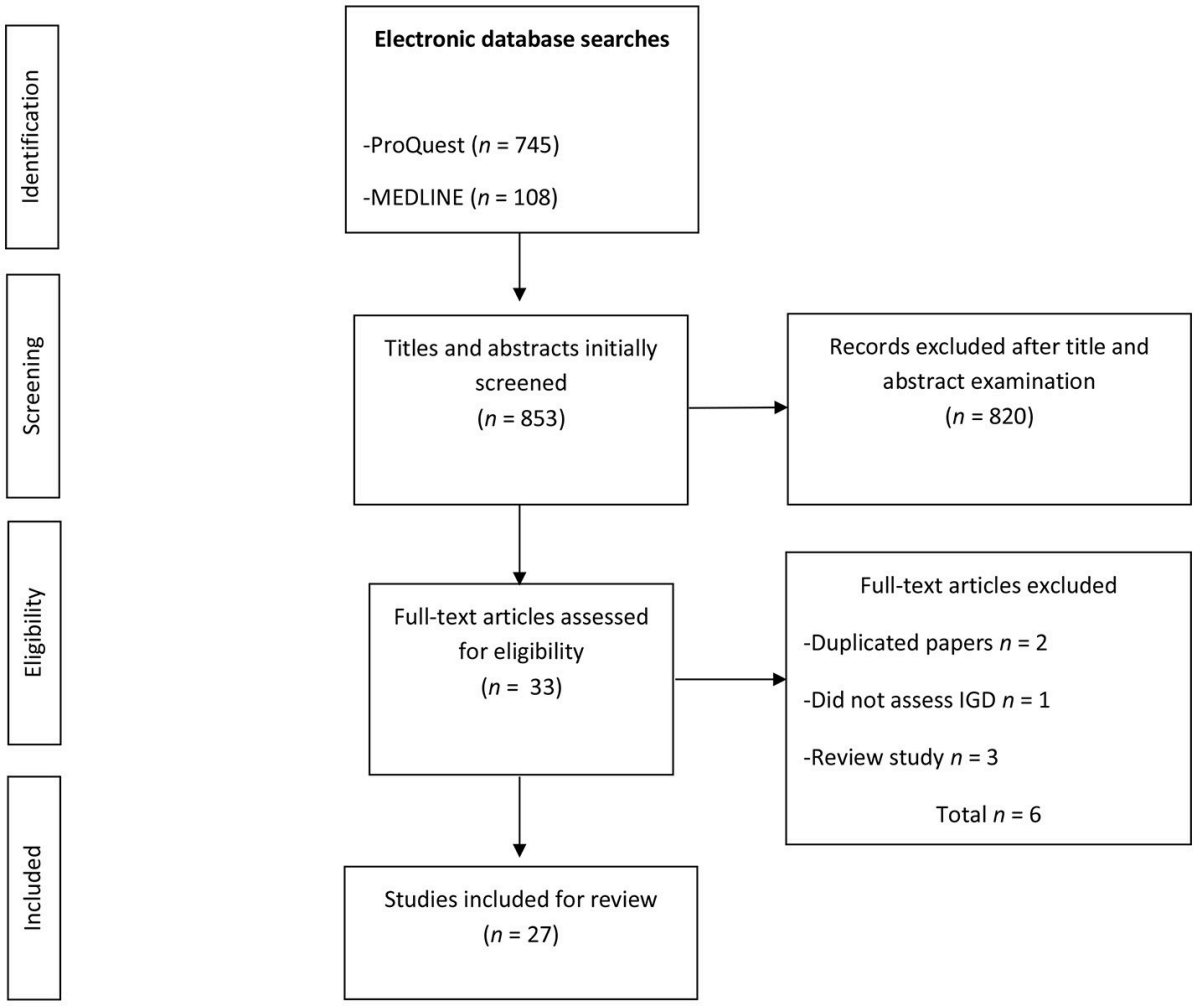
Flow diagram of the study selection process.

A total of 853 studies (ProQuest *n* = 745; MEDLINE *n* = 108) were initially identified, with the search performed on the ProQuest website yielding the following results: ProQuest Psychology Journals *n* = 524; PsycARTICLES *n* = 115; PsycINFO *n* = 106; Applied Social Sciences Index and Abstracts *n* = 0; and ERIC *n* = 0. All 853 papers had their titles and abstracts screened, resulting in the exclusion of 820 papers that were of no relevance for the present review, leaving 33 studies which were eligible for further review. Of these, six papers had to be further excluded because they were either duplicates (*n* = 2), did not assess IGD (*n* = 1), or review papers (*n* = 3). A total of 27 studies were deemed eligible for further analysis as they met the inclusion criteria. The selection process is detailed in the flow chart in Figure 1.

### Functional magnetic resonance imaging (fMRI)

With fMRI, changes in the levels of blood oxygen (BOLD) in the brain are measured because they denote neuronal activity. The ratio of oxyhemoglobin (i.e., hemoglobin which contains oxygen in the blood) to deoxyhemoglobin (i.e., hemoglobin which has released oxygen) in the brain is measured as blood flow in “active” brain areas increases to transport more glucose, whilst transporting additional oxygenated hemoglobin molecules. Measuring this metabolic activity in the brain allows for finer and more detailed imaging of the brain relative to structural MRI. Moreover, the benefits of fMRI comprise the speed of brain imaging, spatial resolution, and no possible health risk in comparison to PET scans (26). A total of four studies were identified that used fMRI in the study of IGD (27–30). The details of these studies are presented in Table 1 below.

**Table 1:** Functional magnetic resonance imaging (fMRI) studies of Internet Gaming Disorder (IGD).

**Author**	**Sample**	**Aims**	**Findings**
Ding et al. (28)	*N* = 34 adolescents recruited from a mental health center in China (50 male; mean age = 16.4, *SD* = 3.2 years)	To assess whether sub-facets of trait impulsivity are linked to brain regions associated with impaired impulse inhibition in individuals with IGD	PFC involved in circuit modulating impulsivity. Impaired PFC function related to high impulsivity in adolescents with IGD, and may contribute to IGD process
Sun et al. (30)	*N* = 39 adolescents and adults with IGD recruited form mental health center, and healthy controls in China (83% male; mean age = 20.5, *SD* = 3.55 years)	To investigate whether diffusional kurtosis imaging (DKI) can be used to detect changes in gray matter (GM) in individuals with IGD	DKI can detect subtle differences in GM microstructure between IGD and healthy individuals. DKI model can provide sensitive imaging biomarkers for assessing IGD severity.
Dieter et al. (27)	*N* = 32 adults with IGD recruited in mental health center, and healthy controls in Germany (91% male; mean age = 26.7, *SD* = 6.3 years)	To measure psychological and neurobiological correlates of relationship between avatar and concepts of self and ideal self in individuals with IGD	Disordered gamers identify significantly more with their avatar than non-disordered individuals. Avatar may replace gamers' ideal self-whilst addiction develops.
Luijten et al. (29)	*N* = 34 male gamers in the Netherlands (mean age = 20.8, *SD* = 3.1 years)	To assess cognitive control deficits in individuals with IGD (e.g., inhibitory control, error processing, attention control)	Reduced inhibitory control, but error processing and attention control normal.

Taken together, the fMRI studies using adolescent samples in China diagnosed with IGD (28, 30) suggested that there were differences between these individuals in comparison to healthy controls with regards to their neurobiology. Specifically, adolescents with IGD were found to have a higher activity in the superior medial frontal gyrus, right anterior cingulate cortex (ACC), right superior and middle frontal gyrus, the left inferior parietal lobule, the left precentral gyrus, and the left precuneus and cuneus, indicating worse response-inhibition and impaired prefrontal cortex (PFC) functioning (28) given previous research as shown that the left frontoparietal network is responsible for response inhibition (31). There was less activity in the bilateral middle and inferior temporal gyri which are responsible for visual processing (such as face recognition), and the right superior parietal lobule (responsible for spatial orientation), suggesting decreased visual and auditory functioning (28). Another fMRI study included in this review included male gamers in the Netherlands (29) and used Go-NoGo and Stroop tasks to assess impulsivity and inhibitory control, finding that problem video game players have lower brain activity in the left inferior frontal gyrus, right inferior parietal lobe in comparison to matched casual gaming controls, indicating that problem gamers have a lower inhibitory control [similar to the results regarding impaired inhibitory control outlined in Ding et al.'s study (28)], with no differences found in attention control and error processing.

Moreover, diffusional kurtosis imaging (measuring water diffusion processes in the brain to assess microstructures) and voxel-based morphometry indicated that adolescents with IGD had lower kurtosis parameters in gray matter (GM) in various neuronal areas, whilst their GM volume in the temporal and parahippocampal gyri was higher, and lower in their left precentral gyrus. Based on the differences found in the assessed mean kurtosis metrics (i.e., water diffusion) between Internet gaming addicts and healthy controls and the brain areas detailed above (30), it appears there are significant differences in the microstructure of the brain between these groups, pointing to a particular IGD pathophysiology (30). [For a detailed representation of the peak MNI coordinates of the voxel and cluster analysis of this study, please refer to the summary provided regarding the MK changes, differences in axial and radial kurtosis between the Internet gaming addiction and the control groups (Sun et al., pp. 48ff.)].

The final fMRI study using adult players of Massively Multiplayer Online Role-Playing Games (MMORPGs) with IGD in Germany (27) showed that they identify with their in-game avatar (i.e., their virtual character), which leads to activation of brain areas associated with self-identification and self-concept-relate processing, i.e., the left angular gyrus, suggesting avatar-identification may be a consequence of compensating for social anxiety, resulting in developing IGD.

### Resting state magnetic resonance imaging (rsfMRI)

rsfMRI is a subtype of fMRI which measures blood oxygen levels (BOLD) to assess brain activity whilst the subject is in a resting state (i.e., not engaged in a specific activity). The aim is to investigate whether there are differences in brain function in individuals with particular conditions in comparison to healthy controls (32). In the present review, a total of seven studies used rsMRI to study IGD were included (33–39). Study details are provided in Table 2.

**Table 2:** Resting state magnetic resonance imaging (rsfMRI) studies of Internet Gaming Disorder (IGD).

**Author**	**Sample**	**Aims**	**Findings**
Xing et al. (36)	*N* = 34 adolescents in China (61% male; mean age = 19.1, *SD* = 0.7 years)	To assess the relationship between the salience network and cognitive control in adolescents with IGD	Right salience network associated with impaired executive function. Structural connectivity differences between adolescents with IGD and healthy controls.
Yuan et al. (38)	*N* = 87 adolescents and young adults in China (75% male; mean age = 19, *SD* = 1.4 years, range = 15–23)	To assess differences in striatum volume and resting-state functional connectivity (RSFC) networks between individuals with IGD and healthy controls	Differences in striatum volume and frontostriatal circuits RSFC between individuals with IGD and healthy controls. Cognitive control deficits in IGD correlated with reduced frontostriatal RSFC strength.
Yuan et al. (33)	*N* = 33 young male gamers and non-gamers in Germany (mean age = 25.5, *SD* = 4.2, range = 18–34)	To assess whether World of Warcraft layers have deficient reward system	Evidence for reward system deficiency in frequent online gamers, including significantly decreased neural activation during anticipation of small and large monetary rewards in ventral striatum
Lin et al. (34); Lin et al. (35)	*N* = 52 male young individuals in China (mean age = 22.2, *SD* = 3.1 years)	To assess abnormal spontaneous brain activity in IGD with low-frequency fluctuation (fALFF) at different frequency bands	Individuals with IGD had lower fALFF values in superior temporal gyrus and higher fALFF values in cerebellum
Wang et al. (39)	*N* = 41 adolescents in China (mean age = 16.9, *SD* = 2.7 years; range = 14–17)	To assess interhemispheric resting state functional connectivity of individuals with IGD using voxel-mirrored homotopic connectivity (VMHC)	Individuals with IGD had decreased VMHC between orbital part of left and right superior, middle and inferior frontal gyrus

Taken together, the rsfMRI studies identified in the present review suggest individuals with IGD have an impaired cognitive control [(34)–(36, 38, 39)], and a deficiency in their ventral striatum reward system (33). Cognitive control in IGD individuals was assessed using a color-word Stroop task, decreased fractional anisotropy (FA) in the right salience network, indicating reduced fiber density, axonal diameter, and myelination in white matter (WM), which may explain problem in regulating the salience network in individuals with IGD that may be associated with impaired cognitive control (36). Decreased WM density in the inferior frontal gyrus, insula, amygdala and anterior cingulate have been demonstrated in individuals with Internet gaming addiction relative to healthy controls, indicating decreased capacities of decision-making, behavioral inhibition and emotion regulation in the IGA group (34). In addition to this, it has been shown that individuals with IGD have decreased fractional amplitudes of low-frequency fluctuation (measuring local brain activity which has been linked to psychiatric disorders) in the cerebellum and increased values in the superior temporal gyrus, suggesting impaired executive function, working memory and decision-making in IGD subjects relative to healthy controls, but also more brain activity that may be associated with increased sensory-motor coordination in IGD (35). Research has also indicated an increased volume of the right caudate and nucleus accumbens (driving the experience of pleasure in the human brain) and reduced strength of resting state functional connectivity in the PFC, tied to decreased cognitive control, similar to these found in substance-related disorders (38). Furthermore, research has found that individuals with IGD have decreased voxel-mirrored homotopic connectivity (measuring the connectivity between brain hemispheres) between the left and right superior frontal gyrus, frontal and middle frontal gyrus, indicating reduced interhemispheric communication in the brain of IGD individuals relative to healthy controls, impacting decision-making, craving and inhibitory errors (39). Moreover, it has been found that individuals who frequently play MMORPGs such as World of Warcraft have a lower physiological responsiveness in the ventral striatum when anticipating monetary rewards with ventral striatum activity differing both with task-based as well as resting-state fMRI, with the deficient sensitivity to reward predisposing individuals to excessive gaming (rather than reward system deficiency being the result of excessive gaming) (33).

### Voxel-based morphometry (VBM)

VBM is a useful technique for understanding IGD as it helps characterize subtle structural changes in the brain without the need of prior knowledge (40). This is especially important given videogame use can affect brain functioning in various ways that may result in changes at the behavioral and cognitive levels (41). This subsection will briefly outline some of the key findings obtained from IGD studies using VBM, and more information is provided in Table 3.

**Table 3:** Voxel-based morphometry (VBM) studies of Internet Gaming Disorder (IGD).

**Author**	**Sample**	**Aims**	**Findings**
Lee et al. (42)	*N* = 61 adolescents and young adults in South Korea (100% male; mean age = 23.5, *SD* = 2.7 years, range = 18–28 years)	To identify gray matter (GM) changes associated with IGD and assess difficulties in executive control by evaluating impulsivity	IGD subjects showed smaller GM volume in brain areas related to executive control. The GM volume in the anterior cingulate cortex and the supplementary motor area were negative associated to impulsivity
Du et al. (43)	*N* = 52 adolescents and young adults in China (100% male; mean age = 17, *SD* = 3 years)	To investigate potential altered structural correlates of impulsivity in IGD adolescents compared to healthy controls	IGD individuals presented with dysfunction in different brain areas involved in the behavior inhibition, attention and emotion regulation
Ko et al. (44)	*N* = 60 adolescents and young adults in Taiwan (100% male; mean age = 23.6, *SD* = 2.5 years)	To evaluate GM density and functional connectivity (FC) in individuals with IGD	IGD individuals showed altered GM density over the amygdala. Further analysis of the amygdala indicated impaired FC to the frontal lobe
Jin et al. (45)	*N* = 46 young adults in China (65% male; mean age = 19.1, *SD* = 1.1 year)	To assess the abnormal structural resting-state properties of several frontal regions in individuals with IGD	IGD individuals showed significant decreased GM volume in the prefrontal cortex (PFC) regions including the bilateral dorsolateral prefrontal cortex (DLPFC), orbitofrontal cortex (OFC), anterior cingulate cortex (ACC), and the right supplementary motor area (SMA)
Weng et al. (46)	*N* = 34 adolescents in China (82% female; mean age = 16.3, *SD* = 3.0 years)	To investigate the differences in the brain morphology between IGD subjects and healthy controls, and to explore the neural possible mechanism of IGD	IGD individuals showed significant GM atrophy in the right OFC, bilateral insula, and right SMA. Overall, microstructure abnormalities of GM and white matter (WM) were found in IGD subjects
Wang et al. (39)	*N* = 56 adolescents in China (67% male; mean age = 18.8, *SD* = 1.3 year)	To investigate cognitive control function and potential alteration of brain GM volume in IGD individuals	GM volume of the bilateral ACC, precuneus, SMA, superior parietal cortex, left DLPFC, left insula, and bilateral cerebellum decreased in IGD individuals in comparison to healthy controls
Lin et al. (34)	*N* = 71 young adults in China (100% male; mean age = 22.2, *SD* = 3.1 years)	To assess if IGD contributes to cerebral structural changes by examining GM and WM density changes in IGD individuals	IGD individuals showed significant lower GM and WM density in several areas of the brain involved in decision-making, behavioral inhibition, and emotional regulation

Lee et al. (42) utilized VBM to investigate the association between GM abnormalities and impulsivity in IGD, and found that IGD subjects exhibited smaller GM volume in brain regions related to executive control, such as the ACC and the supplementary motor area (SMA). It was also found that GM volumes in the ACC and the SMA were negatively associated with impulsiveness, and that IGD subjects exhibited smaller GM volume in the lateral prefrontal and parietal cortices comprising the left ventrolateral PFC and the left inferior parietal lobule when compared to healthy controls. Lee et al. (42) also found that GM volumes in the left ventrolateral PFC were negatively correlated with lifetime usage of videogames. Similarly, further research showed links between GM and impulsivity in IGD individuals. More specifically, Du et al. (43) found that IGD individuals present with higher levels of impulsivity associated with GM volume of the right dorsomedial prefrontal cortex (DMPFC), the bilateral insula and the orbitofrontal cortex (OFC), the right amygdala and decreased left fusiform gyrus. Taken together, these findings suggest GM abnormalities in areas related to executive control may contribute to greater impulsivity in young male adults with IGD, and that dysfunction of these brain areas involved in behavior inhibition, attention and emotion regulation might contribute to impulse control problems in adolescents with IGD (44).

Further research showed that GM density of the bilateral amygdala decreased and the connectivity between the PFC/insula and the amygdala increased in IGD individuals, which suggests emotion dysregulation (44). Furthermore, the altered correlations between impulsivity and GM volume in the DMPFC, OFC, insula, amygdala and the fusiform in IGD adolescents indicate that dysregulation in the brain networks involved in behavior inhibition, attention and emotion regulation might contribute to higher impulsivity levels in adolescents presenting with IGD.

VBM research has helped identifying specific brain regions with GM changes in IGD. Jin et al. (45) found that IGD adolescents showed decreased GM volume in the frontal regions including the bilateral dorsolateral PFC, OFC, ACC, the right SMA and cerebellum after controlling for age and gender effects. These findings are in line with previous studies suggesting GM deficits in the OFC can occur in IGD individuals (46), the involvement of several PFC regions and related PFC –striatal circuits in the process of IGD, and IGD may share similar neural mechanisms with substance dependence at the circuit level.

VBM research has also identified potential detrimental effects of IGD on cognitive control functioning. Wang et al. (39) reported that GM volume of the bilateral ACC, precuneus, SMA, superior parietal cortex, left dorsal lateral PFC, left insula, and bilateral cerebellum decreased significantly in IGD individuals. This study suggests that the alteration of GM volume is associated with performance change of cognitive control in adolescents with IGD, highlighting substantial brain image effects induced by IGD.

Previous VBM research has reported abnormal GM and WM volume in IGD. Lin et al. (34) found that IGD individuals exhibited significantly lower GM density in the bilateral inferior frontal gyrus, left cingulate gyrus, insula, right precuneus, and right hippocampus. It was also found that IGD individuals showed significantly lower WM density in the inferior frontal gyrus, insula, amygdala, and anterior cingulate than healthy controls (34). These findings converge with those reported in earlier studies where IGD subjects were shown to present smaller insular GM density [e.g., (46, 47)], and IGD can negatively affect processes involved in decision-making, behavioral inhibition and emotion.

Overall, VBM research has been helpful in demonstrating potential structural brain changes of IGD individuals. Many of the brain regions found to be altered in IGD individuals have been previously linked to functions contributing to the development of addictive or compulsive behaviors (48). For example, decreased OFC thickness has been identified in individuals with substance-use disorders and behavioral addictions, further implying the development of IGD may involve brain regions similar to those involved in these conditions (49, 50). Although some of the studies reported found changes across different brain regions, these discrepancies help illustrate different ways in which IGD can affect overall brain functioning and the changes it may produce at the behavioral and cognitive level (41), further highlighting the complexity of the phenomenon. Moreover, given that many of the VBM studies reviewed were conducted in adolescent samples and that their brain is still developing, the results reported may not be generalizable across all age groups. One potential avenue to control for this would be to conduct similar studies in samples of children and adults to compare the findings obtained.

### Positron emission tomography (PET)

PET has been utilized to demonstrate that dopamine is released in the human striatum during videogame play, and that playing videogames can lead to significant changes in brain chemistry similar to pharmacologically induced changes (51). The PET studies are summarised in Table 4. Much evidence has implicated the dopaminergic system in the regulation of rewarding behaviors and behavioral addictions, such as IGD (52, 53).

**Table 4:** Positron emission tomography (PET) studies of Internet Gaming Disorder (IGD).

**Author**	**Sample**	**Aims**	**Findings**
Park et al. (54)	*N* = 20 young adults in South Korea (100% male; mean age = 24.7, *SD* = 2.4 years)	To investigate the differences in regional cerebral glucose metabolism at resting state in IGD individuals	IGD individuals showed greater impulsivity and severity of IGD and impulsiveness were associated. IGD individuals had increased glucose metabolism in the orbitofrontal cortex (OFC), striatum, and sensory regions that are implicated in impulse control, reward processing, and somatic representation of previous experiences
Tian et al. (55)	*N* = 26 adolescents and young adults in China (100% male; mean age = 23.5, *SD* = 2.6 years)	To assess brain dopamine D2 (D_2_)/Serotonin 2A (5-HT_2A_) receptor function and glucose metabolism in IGD individuals	IGD individuals showed decreased glucose metabolism in the prefrontal, temporal, and limbic systems. Further dysregulation of D_2_ receptors was found in the striatum and associated to years of IGD

In a ^18^F-fluorodeoxyglucose PET study conducted by Park et al. (54) using a male sample of nine healthy controls and 11 IGD gamers, the authors found greater impulsiveness in IGD players in comparison to healthy controls. Additionally, the imaging data showed IGD gamers had significantly increased glucose metabolism in the right middle orbitofrontal gyrus, left caudate nucleus, and right insula, and decreased metabolism in the bilateral postcentral gyrus, left precentral gyrus, and bilateral occipital regions compared to the control group. In summary, these findings suggest that IGD may share psychological and neural mechanisms with other types of impulse control disorders and substance/nonsubstance-related addiction experiences.

Further research using PET has been carried out in an attempt to shed light on the neurobiological mechanisms of IGD. Tian et al. (55) investigated brain dopamine D_2_ (D_2_)/Serotonin 2A (5-HT_2A_) receptor function and glucose metabolism and whether there was an association between D_2_ receptor and glucose metabolism in a sample of 12 drug-naïve adult males meeting the criteria for IGD and 14 healthy controls using PET and ^11^C-N-methylspiperone to assess the availability of D_2_/5-HT_2A_ receptors and with ^18^F-fluorodeoxyglucose to assess regional brain glucose metabolism, a marker of brain function. The findings o suggested IGD individuals presented with significantly decreased glucose metabolism in the prefrontal, temporal, and limbic systems. Additionally, dysregulation of D_2_ receptors was observed in the striatum and associated with history of excessive videogame play. Further, low levels of D_2_ receptors in the striatum were significantly associated with decreased glucose metabolism in the OFC. Taken together, these findings suggest D_2_/5-HT_2A_ receptor-mediated dysregulation of the OFC underlies a mechanism for loss of control and compulsive behavior in IGD individuals.

Although there is a general scarcity of PET studies on IGD, regarding the imaging techniques utilized, fMRI is preferable to PET because it does not require exposing individuals to radiation (56). However, advantages of PET studies may include its usefulness to ascertain the efficacy of pharmacotherapy and predict treatment outcomes (57).

### Electroencephalography (EEG)

Studies using EEG are employed to detect neural activity from the underlying cortical areas (anterior, posterior, right, and left) in an individual's cerebral cortex using electrodes attached to the scalp. Using this technique, voltage fluctuations (i.e., current flow produced by excitation of neuronal synapses) are measured between pairs of electrodes (58). More specifically, the relationships between an individual's brain and behavior are assessed via electrophysiological neuronal responses to stimuli (59). However, when compared to other neuroimaging techniques (such as fMRI) the spatial resolution in the subcortical areas is poorer. Up to 2013, most of the published studies utilizing EEG [e.g., (60–64)] assessed young adult males with Internet addiction rather than IGD, although the samples used included gamers. Regarding more recent IGD studies using EEG, the main types of study comprise studies examining (i) excessive and addictive gaming, (ii) gaming addiction and other comorbid disorders, and (iii) gaming addiction (miscellaneous). The included studies are presented in Table 5.

**Table 5:** EEG studies examining gaming addiction/Internet Gaming Disorder.

**Author**	**Sample**	**Aims**	**Findings**
Littel et al. (65)	25 excessive gamers (mean age 20.52 years; *SD* = 2.95) compared to 27 non-excessive gamers (mean age 21.42 years; *SD* = 2.59) in The Netherlands (100% male)	To investigate response inhibition and error-processing among excessive gamers compared to casual gamers utilizing the Go/NoGo paradigm	Excessive gamers had poorer error-processing and displayed less inhibition compared to controls
Duven et al. (66)	14 pathological gamers (mean age 24.29 years; *SD* = 5.84) compared to 13 casual gamers (mean age 23.31 years; *SD* = 3.01) in Germany (100% male)	To investigate whether there is enhanced motivational attention or tolerance effects in IGD patients compared to casual gamers	An attenuated P300 for IGD patients in response to rewards compared to controls
Park et al. (67)	26 patients with IGD (20 males; mean age 23.04 years; *SD* = 4.15) compared to 23 healthy controls (20 males; mean age 25.04 years; *SD* = 4.29) in South Korea	To examine dysfunctional information processing among individuals with IGD compared to controls	Those with IGD demonstrated a significant reduction in response to the deviant tones in the P300 amplitudes at the midline centro-parietal electrode regions
Kim et al. (68)	20 patients with IGD (mean age 22.71 years; *SD* = 5.47) compared to 29 healthy controls (mean age 23.97 years; *SD* = 4.36) in South Korea (100% male)	To locate bio-markers associated with IGD compared to controls	Those with IGD showed increased resting-state EEG activity at baseline (delta and theta bands)
Kim et al. (69)	27 patients with IGD (24 males; mean age 26.5 years; *SD* = 6.1) compared to 24 with Obsessive-Compulsive Disorder (19 males; mean age 25.0 years; *SD* = 5.7), and 26 healthy controls (18 males; mean age 24.7 years; *SD* = 4.7) in South Korea	To compare the neurophysiological correlates of altered response inhibition among individuals with IGD and obsessive-compulsive disorder (OCD).	The IGD group demonstrated a delayed NoGo-N2 latency at the central electrode site compared to controls.
Son et al. (70)	34 patients with IGD (mean age 22.71 years; *SD* = 5.47) compared to 17 with Alcohol Use Disorder (mean age 29.71 years; *SD* = 4.88), and 29 healthy controls (mean age 23.88 years; *SD* = 4.66) in South Korea (100% male)	To compare the resting-state QEEG patterns among those with IGD, Alcohol Use Disorder, and healthy controls	IGD group had lower absolute beta power than the other two groups. No significant correlations between the IGD severity and QEEG were found.
Park et al. (48)	16 adolescents with IGD+ADHD (mean age 14.6 years; *SD* = 1.9) compared to 15 adolescents with ADHD (mean age 13.7 years; *SD* = 0.8), and 15 adolescent healthy controls (mean age 14.4 years; *SD* = 1.7) in South Korea (100% male)	To compare adolescent males with ADHD and IGD, male ADHD-only, and a male control group using QEEG	Compared to the ADHD-only group, the IGD/ADHD group had lower relative delta power and greater relative beta power in temporal regions
Youh et al. (71)	14 patients with IGD and Major Depressive Disorder (MDD; mean age 20.00 years; *SD* = 5.9) compared to 15 patients with MDD (mean age 20.3 years; *SD* = 5.5) in South Korea (100% male)	To compare the neurobiological differences between IGD+MDD patients and MDD patients using QEEG	Compared to those with MDD-only, inter-hemispheric coherence value for the alpha band between Fp1–Fp2 electrodes was significantly lower in those with IGD+MDD
Peng et al. (72)	16 patients with IGD (13 males; mean age 20.75 years; *SD* = 0.36) compared to 15 healthy controls (12 males; mean age 20.25 years; *SD* = 0.4) in China (100% male)	To examine the unconscious processing of facial expressions among those with IGD compared to controls using EEG	Those with IGD exhibited decreased amplitudes in ERP component N170 in response to neutral expressions compared to happy expressions in the happy–neutral expressions context

#### Excessive and addictive gaming

In the first study to actually include a sample specified as gamers rather than Internet addicts, Littel et al. (65) investigated response inhibition and error-processing. ERPs of 25 excessive gamers were compared to a control group utilizing the Go/NoGo paradigm. Compared to the control group, excessive gamers had poor error-processing (as indicated by reduced fronto-central ERN amplitudes following incorrect trials in the Go/NoGo task). Moreover, the excessive gamers displayed less inhibition on both behavioral and self-report measures, and results were similar to those with impulse control disorders and substance dependence. The authors speculated that poor error processing, trait impulsivity, and diminished behavioral response inhibition may underlie IGD.

A study by Duven et al. (66) examined whether enhanced motivational attention or tolerance effects are present in IGD patients. IGD patients (*n* = 14) and a control group played a videogame during the recording of ERPs to assess reward processing. The findings demonstrated an attenuated P300 for IGD patients in response to rewards compared to controls. It was also reported that among IGD patients, the latency of N100 was prolonged and the amplitude of N100 was increased. The authors concluded that when playing videogames, tolerance effects are present in IGD patients.

Park et al. (67) used EEG to examine dysfunctional information processing among individuals with IGD. More specifically, they investigated differences in the P300 component of the ERP while participants performed an auditory oddball task. Compared to controls, those with IGD demonstrated a significant reduction in response to the deviant tones in the P300 amplitudes at the midline centro-parietal electrode regions. The authors also reported a negative correlation between IGD severity and P300 amplitudes. It was concluded that the reduced P300 amplitudes may be a neurobiological marker for IGD.

Another study using EEG to try and locate bio-markers associated with IGD was that carried out by Kim et al. (68). The study compared 20 IGD patients with healthy controls over a 6-month period. Using resting-state EEG, participants were scanned prior to and after treatment. Those with IGD showed increased resting-state EEG activity at baseline (delta and theta bands). After 6 months of treatment, increased delta band activity was normalized and significantly correlated with a reduction in IGD symptoms. It was also reported that higher absolute theta activity at baseline predicted a greater improvement in IGD addiction symptoms following treatment. The authors argued that the increased slow-wave activity represented a state neurophysiological marker for those with IGD.

#### Gaming addiction and other comorbid disorders

Kim et al. (69) compared the neurophysiological correlates of altered response inhibition among individuals with IGD and obsessive-compulsive disorder (OCD). A total of 27 IGD patients, 24 OCD patients, and 26 healthy controls participated in a Go/NoGo task while undergoing EEG. The groups were compared on the N2-P3 complexes elicited during Go and NoGo task. The IGD group demonstrated a delayed NoGo-N2 latency at the central electrode site compared to controls. OCD patients had a smaller NoGo-N2 amplitude at the frontal electrode site than those with IGD. The authors concluded that prolonged NoGo-N2 latency may be as a marker of trait impulsivity in IGD and that reduced NoGo-N2 amplitude may be a differential neurophysiological feature between OCD from IGD in regard to compulsivity.

Son et al. (70) compared the resting-state QEEG patterns among those with IGD (*n* = 34), alcohol use disorder (AUD; *n* = 17), and healthy controls (*n* = 25). Results demonstrated that the IGD group had lower absolute beta power than the other two groups. The AUD group had higher absolute delta power than the two other groups. No significant correlations between the IGD severity and QEEG were found. The authors suggested that lower absolute beta power may be a potential trait marker of IGD and that IGD was neurophysiologically distinct from AUD.

In a study by Park et al. (48), the authors noted that IGD is often comorbid with attention deficit hyperactivity disorder (ADHD). Using quantitative electroencephalogram (QEEG) they compared three adolescent groups: males with ADHD and IGD (*n* = 16), male ADHD-only (*n* = 15), and a control group (*n* = 15). Amongst other findings, results showed that compared to the ADHD-only group, the (i) IGD/ADHD group had lower relative delta power and greater relative beta power in temporal regions, (ii) intra-hemispheric coherence values for the bands between P4–O2 electrodes (i.e., delta, theta, alpha, and beta bands) were higher in IGD/ADHD group, and (iii) intra-hemispheric coherence values for the theta band between Fz–Cz and T4–T6 electrodes were higher in IGD/ADHD group. The authors concluded that ADHD adolescents appear to continuously play online videogames to unconsciously enhance attentional ability. They also speculated that “*repetitive activation of brain reward and working memory systems during continuous gaming may result in an increase in neuronal connectivity within the parieto-occipital and temporal regions for the ADHD/IGD group”* (p. 514).

Youh et al. (71) noted that IGD is comorbid with major depressive disorder (MDD). In a study utilizing QEEG, they compared the neurobiological differences between MDD without comorbidity (MDD-only; *n* = 15) and MDD comorbid with IGD (MDD+IGD; *n* = 14). EEG coherences were measured using a 21-channel digital EEG system and computed to assess synchrony in the frequency ranges of alpha and beta between 12 electrode site pairs. The results demonstrated that compared to those with MDD-only (i) inter-hemispheric coherence value for the alpha band between Fp1–Fp2 electrodes was significantly lower in those with IGD, (ii) intra-hemispheric coherence value for the alpha band between P3–O1 electrodes was higher in those with IGD, and (iii) intra-hemispheric coherence values for the beta band between F8–T4, T6–O2, and P4–O2 electrodes were higher in those with IGD. The authors concluded that excessive online gaming may lead to increased intra-hemisphere connectivity in the fronto-temporo-parieto-occipital areas.

#### Gaming addiction (miscellaneous)

One of the more unusual studies examining IGD with EEG is a study by Peng et al. (72) who examined the unconscious processing of facial expressions among those with IGD. The authors claimed that “*IGD is characterized by impairments in social communication and the avoidance of social contact. Facial expression processing is the basis of social communication”* (p. 1). Consequently, they investigated how those with IGD process facial expressions. To examine the differences between the processing of subliminally presented facial expressions (happy, neutral, sad) with ERPs, those with IGD (*n* = 16) and controls participated in a backward masking task. The findings showed those with IGD were slower than controls in response to both sad and neutral expressions in the sad–neutral context. The ERP results demonstrated those with IGD exhibited “*decreased amplitudes in ERP component N170 (an index of early face processing) in response to neutral expressions compared to happy expressions in the happy–neutral expressions context, which might be due to their expectancies for positive emotional content”* (p. 1). Controls exhibited similar N170 amplitudes in response to both sad and neutral expressions in the sad–neutral expressions context, and happy and neutral expressions in the happy–neutral expressions context. The authors concluded those with IGD have different unconscious neutral facial processing patterns compared to normal controls.

Examining the ten EEG studies as a whole, there is little similarity in any of the 10 studies except that they all have small sample sizes and all found significant differences between those with IGD and the control groups concerning the variable(s) under focus. Two studies reported those with IGD had lower inhibition compared to controls (65, 68) but other than this, no other studies compared the same variables so little can be concluded from EEG studies.

## Discussion

The research of neurobiological correlates in IGD is relevant particularly in light of the National Institute of Mental Health's (NIMH) support for establishing research domain criteria based on which mental disorders should be classified and may offer a solution to the ongoing debates in the IGD field [e.g., (5)]. IGD neuroimaging is a nascent field that is developing at a fast pace, which has been highlighted by the present review. Taken together, the fMRI and rsfMRI studies presented indicate that there appear to be significant neurobiological differences between healthy controls and individuals with IGD. The included studies suggest gaming addicts have worse response-inhibition and emotion regulation, impaired PFC functioning and cognitive control, worse working memory and decision-making capabilities, decreased visual and auditory functioning, and a deficiency in their neuronal reward system. These deficiencies are similar to those found in individuals with substance-related addictions, suggesting that both substance-related and behavioral addictions share common predisposing factors and may be part of an addiction syndrome (73, 74). For example, research in the context of alcohol abuse has found that P300 amplitudes are reduced in individuals who have an increased genetic risk for alcoholism (75, 76). This may suggest that similar findings with reduced P300 amplitudes in individuals with IGD have an elevated genetic risk to developing addiction-related problems. Consequently, future research needs to assess possible genetic vulnerability for developing IGD-related problems to verify such conjecture. However, in the fMRI and rsfMRI studies, no differences were found in attention control and error processing between IGD individuals and healthy controls. Moreover, more brain activity was found in gaming addicts relative to healthy controls, suggesting an increased sensory-motor coordination in IGD. Recent research suggests that regular gaming may have therapeutic benefits and gaming can be used to improve a variety of cognitive and motor skills, and is successfully used in the training of professionals, such as soldiers and surgeons (77).

Despite the invaluable contributions offered by neuroimaging studies on IGD, several limitations potentially compromising the generalizability of the results of these studies need to be highlighted. As the majority of these studies are cross-sectional, it is not possible to ascertain the causal relationships between IGD and the altered structures in the brain reported across these studies, particularly the VBM studies. Future research should adopt other research designs that help overcome these shortcomings. For example, further prospective studies are necessary to understand the roles of altered brain structures in the mechanism of IGD. In addition to this, further studies would benefit from larger sample sizes, as the presently reviewed studies were limited with regards to the number of participants that have been included. Another well-known problem in these studies is the use of generalized Internet addiction assessment tools to assess IGD [see (78), for a review on the topic]. Finally, other major psychiatric disorders were excluded from most VBM studies, thus there is some inherent limitation regarding generalizing the results to subjects with IGD with other substance-use or psychiatric disorders.

Moreover, EEG is commonly used in experimental situations because of its generally non-invasive and unobtrusive nature. Another key strength of EEG studies is that they are all strictly controlled laboratory experiments that can identify causal relationships between the variables assessed. Overall, the EEG findings demonstrate that compared to control groups, gaming addicts have decreased P300 amplitudes and an increased P300 latency (reflecting attention allocation). These differences suggest that those with IGD have an impaired attention capacity or they are unable to adequately allocate attention. Findings of these studies also appear to be similar to EEG studies examining other more traditional addictions, such as those to alcohol and cocaine [e.g., (79–81)]. However, one of the key weaknesses in EEG research is that is unable to provide any direct insights into active transmitter systems of the brain when monitoring brain activity.

In a review of electrophysiological correlates of problematic Internet use, D'Hondt et al. (82) noted that problematic internet use which often includes gaming is particularly associated with a reduction of inhibitory control and an increase in cue-reactivity. The EEG literature demonstrates “*that most studies have found that impaired self-control abilities (i.e., inhibition and error monitoring) are associated with underactivated frontal regions in problematic Internet users”* (p. 64). Furthermore, they noted that some EEG studies in the area demonstrate alterations in the processing of emotional stimuli and Internet-related cues, suggesting that “*both reflective (top-down) and automatic/affective (bottom-up) systems, postulated by dual-process models as being determinants in decision making, are impaired among [problematic internet users”* (p. 64). Overall, the present EEG studies agree with these conclusions because EEG studies reviewed in this section indicate that the brains of those with IGD appear to be less efficient in information processing and response inhibition compared to controls. Consequently, such individuals have low impulse control, use increased cognitive resources to complete specific tasks, and appear to have impaired executive control, again demonstrating similarities with other more traditional addictions (79).

In summary, the presented studies suggest that there may be a particular IGD pathophysiology, in support of the NIMH's advocacy of utilizing RDoC criteria for diagnosing mental disorders (24). Future research should focus on replicating the reported findings in different cultural contexts, in support of a neurobiological basis of classifying IGD and related disorders.

## Author contributions

DK has reviewed, analyzed and written the sections of fMRI and rsfMRI and written the introduction, methods and discussion. MG has reviewed, analyzed and written the section on EEG and contributed to the full manuscript. HP has reviewed, analyzed and written the sections on VBM and PET and contributed to the full manuscript.

### Conflict of interest statement

The authors declare that the research was conducted in the absence of any commercial or financial relationships that could be construed as a potential conflict of interest.
